# Crystal structure determination of two pyridine derivatives: 4-[(*E*)-2-(4-meth­oxy­phen­yl)ethen­yl]-1-methyl­pyridin-1-ium hexa­fluoro-λ^6^-phosphane and 4-{(*E*)-2-[4-(di­methyl­amino)­phen­yl]ethen­yl}-1-phenyl-1λ^5^-pyridin-1-ylium hexa­fluoro-λ^6^-phosphane

**DOI:** 10.1107/S2056989019001403

**Published:** 2019-01-31

**Authors:** J. Arul Martin Mani, M. Mercina, S. Antony Inglebert, P. Narayanan, V. Joseph, P. Sagayaraj

**Affiliations:** a Department of Physics, Loyola College (Autonomous), Chennai 600 034, India; b Department of Physics, A. M. Jain College, Chennai 600 114, India

**Keywords:** crystal structure, cation, anion, disorder, pyridine derivatives, hydrogen bonding, halogen bonding, mol­ecular sheets

## Abstract

The title mol­ecular salts, C_16_H_16_NO^+^·PF_6_^−^, (I), and C_21_H_21_N_2_^+^·PF_6_^−^, (II), are pyridine derivatives. In compound (I), the cation comprises a methyl N-substituted pyridine ring and a meth­oxy-substituted benzene ring connected by a C=C double bond. The F atoms of the PF_6_^−^ anion are disordered over two sets of sites with refined occupancy factors of 0.614 (7):0.386 (7). In compound (II), the cation comprises a pyridine ring attached to unsubstituted phenyl ring and a di­methyl­aniline ring, which are connected by a C=C double bond. The anion is PF_6_^−^. In both salts, the cation adopts an *E* configuration with respect to the C=C bond. The pyridine ring makes a dihedral angle of 9.86 (12)° with the meth­oxy-substituted benzene ring in compound (I) and 11.2 (3)° with the di­methyl­amine-substituted benzene ring in compound (II). In compound (I), the crystal packing is stabilized by weak C—H⋯F inter­molecular inter­actions which result in *R* ^4^_3_(14) ring motifs, forming mol­ecular sheets running parallel to (03). These are further stabilized by weak P—F⋯π interactions. In compound (II), the crystal packing is stabilized by C—H⋯F inter­actions, which result in *R* ^6^_6_(40) ring motifs, forming mol­ecular sheets running parallel to (101) and these are further connected by π–π inter­actions.

## Abstract 2

In both title pyridine derivatives, (I) and (II), the cation adopts an *E* configuration with respect to the C=C. In compound (I), the PF_6_^−^ anion is disordered with occupancy factors of 0.614 (7):0.386 (7). In both the compounds, the crystal packing is stabilized by C—H⋯F inter­molecular inter­actions results into two-dimensional mol­ecular sheets, which are formed by 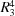
(14) ring motifs in compound (I), 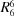
(40) ring motifs in compound (II). In addition to that, the crystal packing is further stabilized by P—F⋯π inter­actions in compound (I) and π–π in compound (II).

## Chemical context   

Stilbene-based compounds are the basic element for a number of biologically active natural and synthetic compounds. These compounds have a wide range of biological activities including anti-inflammatory, anti­cancer, anti­viral, anti­oxidant and more recently neuroprotective effect (Giacomini *et al.*, 2016 ▸). Pyridine and its derivatives play an important role in developing anti­cancer drugs (Ghattas *et al.*, 2017 ▸) and show anti­bacterial activities (Chanawanno *et al.*, 2010 ▸). Pyridine is the parent ring system of a large number of naturally occurring products and important industrial, pharmaceutical and agricultural chemicals. Pyridine derivatives have also shown anti­chagasic activity against Chagas disease, a parasitic infection caused by *Trypanosoma cruzi*, a parasite that is widely spread in central and South America (Dorigo *et al.*, 1993 ▸). The title compounds have been tested for *in vitro* cytotoxicity and anti­cancer activity, using VERO and MCF-7 (breast cancer) cell lines, respectively. The cells were maintained in minimal essential medium supplemented with 10% FBS, penicillin (100 U ml^−1^), and streptomycin (100 microgram ml^−1^) in a humidified atmosphere of 50 microgram ml^−1^ CO_2_ at 310 K.
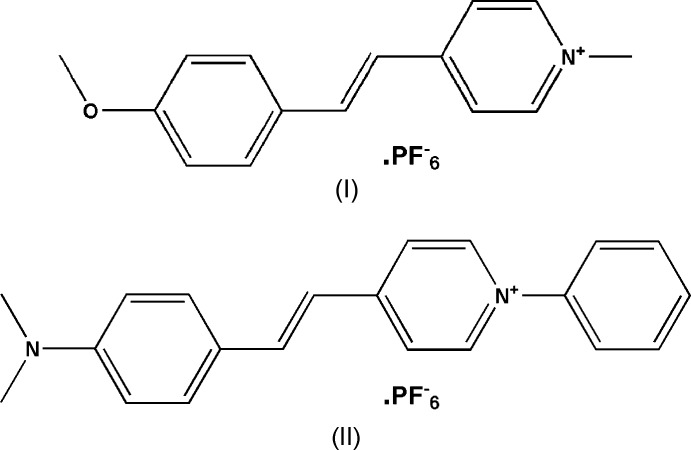


## Structural commentary   

**Figure 1:**
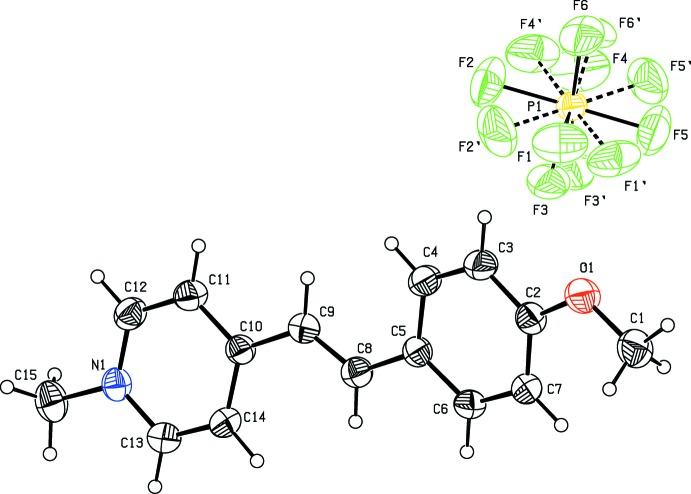
The mol­ecular structure of the title compound (I) with the atom-numbering scheme. Displacement ellipsoids are drawn at the 30% probability level. In the anion, dashed bonds indicate the minor disorder component.

The mol­ecular structures of the title pyridine derivatives [C_16_H_16_NO^+^. PF_6_^−^], (I) and [C_21_H_21_N_2_^+^. PF_6_^−^], (II), are shown in Figs. 1 ▸ and 2 ▸, respectively. In compound (I), the cation comprises a methyl N-substituted pyridine ring (N1/C10–C14) and a meth­oxy-substituted phenyl ring (C2–C7) connected by the C8=C9 bond. The F atoms of the PF_6_^−^ anion are disordered over two sets of sites with refined occupancy factors of 0.614 (7):0.386 (7). In compound (II), the cation comprises a pyridine ring (N2/C7–C11) attached to an unsubstituted phenyl ring (C1–C6) and a di­methyl­amine-substituted pheny ring (C14–C19), connected by the C12=C13 bond. A PF_6_^−^ anion is also present.

In both compounds, the cations adopt an *E* configuration with respect to the C=C bond [C8=C9 = 1.312 (4) Å in compound (I) and C12=C13 = 1.348 (8) Å in compound (II)]. The pyridine ring (N1/C10–C14) makes a dihedral angle of 9.86 (12)° with meth­oxy-substituted benzene ring (C2–C7) in compound (I) whereas in compound (II) the pyridine ring (N2/C7–C11) makes a dihedral angle of 11.2 (3)° with di­methyl­amine-substituted benzene ring (C14–C19). The pyridine ring in compound (II) is inclined to the unsubstituted phenyl ring (C1–C6) by 54.9 (3)°. The meth­oxy group oxygen atom O1 of compound (I) deviates from the benzene ring to which it is attached by 0.0317 (1) Å while the methyl group carbon atom C15 deviates from the benzene ring to which it is attached by 0.022 (3) Å. In compound (II), the methyl­amine group nitro­gen atom (N1) deviates from the benzene ring to which it is attached by 0.017 (5) Å.

In compound (I), the meth­oxy group is (+) anti-periplanar to the phenyl ring (C2–C7), as is evident from the torsion angle C3—C2—O1—C1 of 178.2 (3)°. In compound II, the methyl­amine group is (−) anti-periplanar to the phenyl ring (C14–C19), which is evident from the torsion angle C16—C17—N1—C21 of −173.9 (5)°.

## Supra­molecular features   

**Figure 3:**
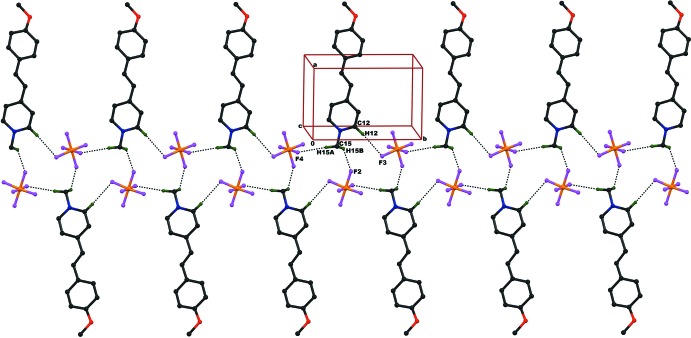
The crystal packing of the title compound (I), viewed along the *a* axis, showing C—H⋯F inter­molecular inter­actions, resulting in *R^4^_3_(14)* ring motifs, which form two-dimensional mol­ecular sheets running parallel to (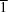
03). Hydrogen atoms not involved in hydrogen bonding have been omitted for clarity.

In the crystal packing of compound (I), the mol­ecules are linked *via* inter­molecular C12—H12⋯F3(−2 + *x*, *y*, −1 + *z*), C15—H15*A*⋯F4(−2 + *x*, −1 + *y*, −1 + *z*) and C15—H15*B*⋯F2(−1 − *x*, −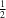 + *y*, *z*) inter­actions (Table 1 ▸), resulting in 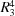
(14) ring motifs, which form mol­ecular sheets lying parallel to (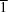
03) (Fig. 3 ▸). The crystal packing is further stabil­ized by P1—F4⋯*Cg*1(−*x*, 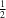 + *y*, −*z*) halogen-bond (XB) inter­actions, where *Cg*1 is the centroid of the pyridine ring (N1/C10–C14).

**Figure 4:**
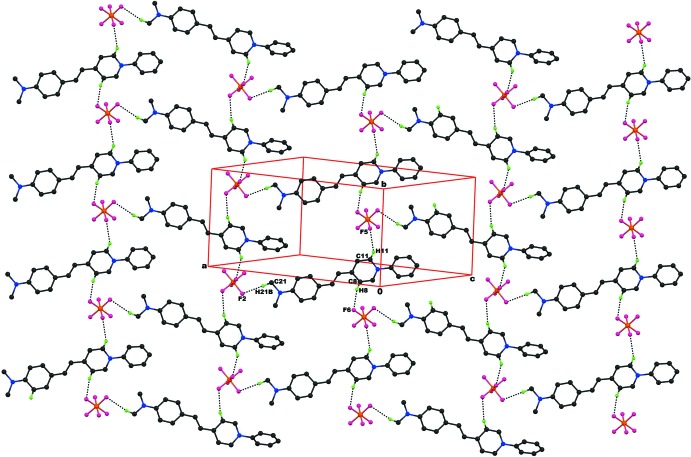
The crystal packing of the title compound (II), viewed along the *b* axis, showing inter­molecular C—H⋯F inter­actions, resulting in 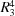
(14) ring motifs, which form mol­ecular sheets lying parallel to (101). Hydrogen atoms not involved in hydrogen bonding have been omitted for clarity.

In the crystal packing of compound (II), intra­molecular C8—H8⋯F6 and inter­molecular C11—H11⋯F5(*x*, 1 + *y*, *z*) and C21—H21*B*⋯F2(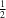 + *x*, −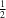 − *y*, −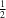 + *z*) inter­actions (Table 2 ▸) result in 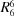
(40) ring motifs and form mol­ecular sheets lying parallel to (101) (Fig. 4 ▸). These mol­ecular sheets are cross-linked by C16—H16⋯F4(*x*, −1 − *y*, −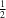 + *z*) inter­actions, resulting in a three-dimensional network. The crystal packing is further stabilized by *Cg*1⋯*Cg*3(*x*, −*y*, −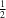 + *z*) inter­actions [centroid–centroid distance = 3.646 (4) Å and inter­planar distance = 3.397 (2) Å], where *Cg*1 is the centroid of the pyridine ring (N2/C7–C11) and *Cg*3 is the centroid of the phenyl ring (C14–C19).

## Database survey   

A search of the Cambridge Structural Database (CSD, V5.39, latest update August 2018; Groom *et al.*, 2016 ▸) found no entry for a hexa­fluoro-λ^6^-phosphane with pyridine derivatives. However, the cationic structures of substituted pyridine derivatives were found, for example, *r*-1,*t*-3-bis­[4-(di­methyl­amino)phen­yl]-*c*-2,*t*-4-bis(pyridin-4-yl)cyclo­butane (Zhang & Zhuang, 2014 ▸) and 4′-hy­droxy-3′-meth­oxy-*N*-methyl4-stil­bazofium tosyl­ate hydrate (Zhang *et al.*, 1997 ▸).

## Synthesis and crystallization   

**Compound (I)**

A solution of *N*-phenyl-4-picolinium chloride (250 mg, 1.10 mmol), 4-(di­methyl­amino) benzaldehyde (363 mg, 2.4 mmol), and piperidine (4 drops) in methanol (20 ml) was heated under reflux for 4 h. The addition of diethyl ether to the deep-red solution yielded a dark precipitate, which was filtered, washed with diethyl ether and dried. This crude chloride salt was metathesized to di­methyl­amino *N*-phenyl stilbazolium hexa­fluoro phosphate (DAPSH) by precipitation from water/aqueous NH_4_PF_6_. A supersaturated solution of DAPSH was prepared using aceto­nitrile as solvent and the solution was filtered into the growth vessel for slow evaporation by covering the vessel with a perforated sheet. Good quality greenish crystals of compound (I) was grown in a period of 15–25 days.

**Compound (II)**

Compound (II) was synthesized by the condensation of 1,4-di­methyl­pyridinium iodide (2.35 g, 10 mmol), methanol (30 ml) and 4-meth­oxy­benzaldehyde (1.36 g, 10 mmol) in the presence of piperidine (0.2 ml). The total mixture was taken in the round-bottom flask (1000 ml capacity) of a Dean–Stark apparatus and refluxed for 1 d and cooled to room temperature. The product 4-meth­oxy-*N*-methyl-4-stilbazolium iodide was filtered and recrystallized from methanol. This product (0.706 g, 2 mmol) was dissolved in 70 ml of millipore water and simultaneously sodium hexa­fluoro­phosphate (0.338 g, 2 mmol) was dissolved in 30 ml of millipore water by heating at 343 K. Both the solutions were stirred for 3 h and mixed. 4-Meth­oxy-*N*-methyl­stilbazolium hexa­fluoro­phosphate (MMSHP) was formed as a yellowish precipitate. A solution of MMSHP and aqueous acetone was prepared with 14.4 g of MMSHP in 200 ml of acetone–water mixed solvent (5:1) and stirred. The clear solution was collected in the growth vessel after filtering it by using 0.2 micrometer porosity millipore filters and the solvent was allowed to evaporate slowly at room temperature. After three weeks, yellowish crystals of compound (II) were harvested.

## Refinement   

Crystal data, data collection and structure refinement details for compounds (I) and (II) are summarized in Table 3 ▸. The positions of the hydrogen atoms were localized from the difference-electron-density maps and their distances were geometrically constrained. The hydrogen atoms bound to the C atoms were treated as riding atoms, with *d*(C—H) = 0.93 and 0.96 Å for aryl and methyl H atoms, respectively, with *U*_iso_(H)= 1.5*U*_eq_(methyl C) and 1.2*U*_eq_(non-methyl C). The rotation angles for methyl groups were optimized by least squares.

## Supplementary Material

Crystal structure: contains datablock(s) I, II, global. DOI: 10.1107/S2056989019001403/lh5891sup1.cif

Structure factors: contains datablock(s) I. DOI: 10.1107/S2056989019001403/lh5891Isup2.hkl

Structure factors: contains datablock(s) II. DOI: 10.1107/S2056989019001403/lh5891IIsup3.hkl

Supporting information file. DOI: 10.1107/S2056989019001403/lh5891Isup4.cml

Supporting information file. DOI: 10.1107/S2056989019001403/lh5891IIsup5.cml

CCDC references: 1893497, 971522

Additional supporting information:  crystallographic information; 3D view; checkCIF report

## Figures and Tables

**Figure 2:**
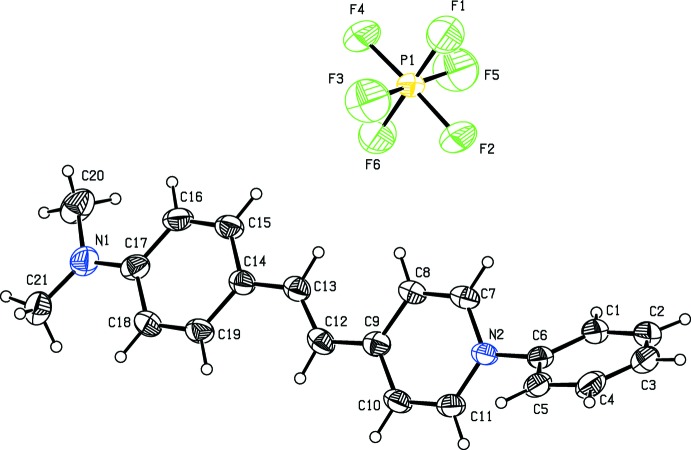
The mol­ecular structure of the title compound (II) with the atom-numbering scheme. Displacement ellipsoids are drawn at 30% probability level.

**Table 1:** Hydrogen-bond geometry (Å, °) for (I)

*D*—H⋯*A*	*D*—H	H⋯*A*	*D*⋯*A*	*D*—H⋯*A*
C1—H1*C*⋯F2′^i^	0.96	2.51	3.220 (8)	131
C12—H12⋯F3^ii^	0.93	2.60	3.509 (8)	165
C12—H12⋯F3′^ii^	0.93	2.53	3.454 (16)	176
C13—H13⋯F5′^iii^	0.93	2.40	3.270 (9)	156
C15—H15*A*⋯F4^iii^	0.96	2.53	3.443 (7)	160
C15—H15*B*⋯F2^iv^	0.96	2.46	3.235 (5)	138
C15—H15*B*⋯F5′^v^	0.96	2.49	3.162 (7)	127

Symmetry codes: (i) 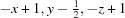; (ii) 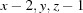; (iii) 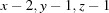; (iv) 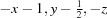; (v) 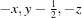.

**Table 2:** Hydrogen-bond geometry (Å, °) for (II)

*D*—H⋯*A*	*D*—H	H⋯*A*	*D*⋯*A*	*D*—H⋯*A*
C8—H8⋯F6	0.93	2.59	3.486 (8)	162
C11—H11⋯F5^i^	0.93	2.55	3.363 (8)	146
C16—H16⋯F4^ii^	0.93	2.59	3.289 (7)	132
C21—H21*B*⋯F2^iii^	0.96	2.64	3.516 (8)	152

Symmetry codes: (i) 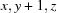; (ii) 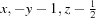; (iii) 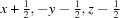.

**Table 3:** Experimental details

	(I)	(II)
Crystal data		
Chemical formula	C_15_H_16_NO^+^·PF_6_^−^	C_21_H_21_N_2_^+^·PF_6_^−^
*M*_r_	371.26	446.37
Crystal system, space group	Monoclinic, *P*2_1_	Monoclinic, *C* *c*
Temperature (K)	296	296
*a*, *b*, *c* (Å)	6.4320 (2), 9.3645 (3), 13.6070 (5)	19.4596 (14), 10.7416 (8), 11.9654 (9)
β (°)	101.868 (2)	125.864 (2)
*V* (Å^3^)	802.06 (5)	2026.9 (3)
*Z*	2	4
Radiation type	Mo *K*α	Mo *K*α
μ (mm^−1^)	0.24	0.20
Crystal size (mm)	0.35 × 0.30 × 0.30	0.35 × 0.30 × 0.30

Data collection		
Diffractometer	Bruker Kappa APEXII CCD	Bruker Kappa APEXII CCD
Absorption correction	Multi-scan (*SADABS*; Bruker, 2008 ▸)	Multi-scan (*SADABS*; Bruker, 2008 ▸)
*T*_min_, *T*_max_	0.921, 0.932	0.933, 0.943
No. of measured, independent and observed [*I* > 2σ(*I*)] reflections	8106, 2867, 2606	13796, 3926, 2895
*R*_int_	0.022	0.024
(sin θ/λ)_max_ (Å^−1^)	0.617	0.617

Refinement		
*R*[*F* ^2^ > 2σ(*F* ^2^)], *wR*(*F* ^2^), *S*	0.032, 0.084, 1.02	0.064, 0.203, 1.07
No. of reflections	2867	3926
No. of parameters	273	255
No. of restraints	140	65
H-atom treatment	H atoms treated by a mixture of independent and constrained refinement	H atoms treated by a mixture of independent and constrained refinement
Δρ_max_, Δρ_min_ (e Å^−3^)	0.15, −0.14	0.49, −0.41
Absolute structure	Flack (1983 ▸), 1198 Friedel pairs	Flack (1983 ▸), 1927 Fridel pairs
Absolute structure parameter	0.08 (11)	0.5 (2)

Computer programs: *APEX2* and *SAINT* (Bruker, 2008 ▸), *SHELXS97* and *SHELXL97* (Sheldrick, 2008 ▸), *ORTEP-3 for Windows* (Farrugia, 2012 ▸), *Mercury* (Macrae *et al.*, 2008 ▸), and *PLATON* (Spek, 2009 ▸).

## supplementary crystallographic information

### 4-[(E)-2-(4-Methoxyphenyl)ethenyl]-1-methylpyridin-1-ium hexafluoro-λ6-phosphane (I) Crystal data

**Table:**

C_15_H_16_NO^+^·PF_6_^−^	*F*(000) = 380
*M**_r_* = 371.26	*D*_x_ = 1.537 Mg m^−^^3^
Monoclinic, *P*2_1_	Mo *K*α radiation, λ = 0.71073 Å
Hall symbol: P 2yb	Cell parameters from 2867 reflections
*a* = 6.4320 (2) Å	θ = 2.7–26.0°
*b* = 9.3645 (3) Å	µ = 0.24 mm^−^^1^
*c* = 13.6070 (5) Å	*T* = 296 K
β = 101.868 (2)°	Block, green
*V* = 802.06 (5) Å^3^	0.35 × 0.30 × 0.30 mm
*Z* = 2	

### 4-[(E)-2-(4-Methoxyphenyl)ethenyl]-1-methylpyridin-1-ium hexafluoro-λ6-phosphane (I) Data collection

**Table:**

Bruker Kappa APEXII CCD diffractometer	2867 independent reflections
Radiation source: fine-focus sealed tube	2606 reflections with *I* > 2σ(*I*)
Graphite monochromator	*R*_int_ = 0.022
ω & φ scans	θ_max_ = 26.0°, θ_min_ = 2.7°
Absorption correction: multi-scan (SADABS; Bruker, 2008)	*h* = −7→7
*T*_min_ = 0.921, *T*_max_ = 0.932	*k* = −9→11
8106 measured reflections	*l* = −16→16

### 4-[(E)-2-(4-Methoxyphenyl)ethenyl]-1-methylpyridin-1-ium hexafluoro-λ6-phosphane (I) Refinement

**Table:**

Refinement on *F*^2^	Hydrogen site location: inferred from neighbouring sites
Least-squares matrix: full	H atoms treated by a mixture of independent and constrained refinement
*R*[*F*^2^ > 2σ(*F*^2^)] = 0.032	*w* = 1/[σ^2^(*F*_o_^2^) + (0.0459*P*)^2^ + 0.106*P*] where *P* = (*F*_o_^2^ + 2*F*_c_^2^)/3
*wR*(*F*^2^) = 0.084	(Δ/σ)_max_ < 0.001
*S* = 1.02	Δρ_max_ = 0.15 e Å^−^^3^
2867 reflections	Δρ_min_ = −0.14 e Å^−^^3^
273 parameters	Extinction correction: SHELXL, Fc^*^=kFc[1+0.001xFc^2^λ^3^/sin(2θ)]^-1/4^
140 restraints	Extinction coefficient: 0.016 (3)
Primary atom site location: structure-invariant direct methods	Absolute structure: Flack (1983), 1198 Friedel pairs
Secondary atom site location: difference Fourier map	Absolute structure parameter: 0.08 (11)

### 4-[(E)-2-(4-Methoxyphenyl)ethenyl]-1-methylpyridin-1-ium hexafluoro-λ6-phosphane (I) Special details

**Table:**

Geometry. All esds (except the esd in the dihedral angle between two l.s. planes) are estimated using the full covariance matrix. The cell esds are taken into account individually in the estimation of esds in distances, angles and torsion angles; correlations between esds in cell parameters are only used when they are defined by crystal symmetry. An approximate (isotropic) treatment of cell esds is used for estimating esds involving l.s. planes.
Refinement. Refinement of F^2^ against ALL reflections. The weighted R-factor wR and goodness of fit S are based on F^2^, conventional R-factors R are based on F, with F set to zero for negative F^2^. The threshold expression of F^2^ > 2sigma(F^2^) is used only for calculating R-factors(gt) etc. and is not relevant to the choice of reflections for refinement. R-factors based on F^2^ are statistically about twice as large as those based on F, and R- factors based on ALL data will be even larger.

### 4-[(E)-2-(4-Methoxyphenyl)ethenyl]-1-methylpyridin-1-ium hexafluoro-λ6-phosphane (I) Fractional atomic coordinates and isotropic or equivalent isotropic displacement parameters (Å^2^)

**Table:**

	*x*	*y*	*z*	*U*_iso_*/*U*_eq_	Occ. (<1)
C1	0.6868 (5)	0.3750 (4)	0.1233 (2)	0.0795 (9)	
H1A	0.7984	0.4199	0.1710	0.119*	
H1B	0.7430	0.3396	0.0679	0.119*	
H1C	0.6290	0.2971	0.1550	0.119*	
C2	0.3506 (4)	0.4329 (3)	0.02002 (19)	0.0562 (6)	
C3	0.1982 (4)	0.5355 (3)	−0.0083 (2)	0.0638 (7)	
H3	0.2204	0.6270	0.0182	0.077*	
C4	0.0127 (4)	0.5051 (3)	−0.07556 (19)	0.0605 (6)	
H4	−0.0885	0.5764	−0.0937	0.073*	
C5	−0.0260 (4)	0.3690 (3)	−0.11688 (16)	0.0494 (6)	
C6	0.1323 (4)	0.2680 (3)	−0.08913 (18)	0.0559 (6)	
H6	0.1122	0.1769	−0.1167	0.067*	
C7	0.3206 (4)	0.2978 (3)	−0.0214 (2)	0.0579 (7)	
H7	0.4246	0.2281	−0.0042	0.069*	
C8	−0.2243 (4)	0.3294 (4)	−0.18350 (15)	0.0536 (5)	
H8	−0.2377	0.2349	−0.2049	0.064*	
C9	−0.3863 (4)	0.4140 (3)	−0.21639 (19)	0.0573 (6)	
H9	−0.3690	0.5098	−0.1986	0.069*	
C10	−0.5907 (4)	0.3729 (3)	−0.27799 (17)	0.0500 (6)	
C11	−0.7440 (4)	0.4771 (3)	−0.3090 (2)	0.0601 (6)	
H11	−0.7139	0.5720	−0.2915	0.072*	
C12	−0.9364 (5)	0.4417 (3)	−0.3646 (2)	0.0613 (7)	
H12	−1.0367	0.5131	−0.3844	0.074*	
C13	−0.8419 (5)	0.2036 (3)	−0.3640 (2)	0.0593 (7)	
H13	−0.8759	0.1099	−0.3836	0.071*	
C14	−0.6466 (5)	0.2330 (3)	−0.3077 (2)	0.0588 (7)	
H14	−0.5494	0.1594	−0.2888	0.071*	
C15	−1.1961 (4)	0.2735 (4)	−0.4540 (2)	0.0741 (8)	
H15A	−1.2061	0.1728	−0.4675	0.111*	
H15B	−1.2142	0.3250	−0.5162	0.111*	
H15C	−1.3048	0.3012	−0.4189	0.111*	
N1	−0.9864 (3)	0.3063 (2)	−0.39178 (14)	0.0543 (6)	
O1	0.5245 (3)	0.4760 (2)	0.08788 (15)	0.0736 (6)	
P1	0.74137 (10)	0.83995 (8)	0.65677 (4)	0.05309 (18)	
F1	0.7553 (11)	0.7695 (5)	0.7626 (3)	0.1143 (17)	0.614 (7)
F2	0.5074 (6)	0.8863 (7)	0.6568 (4)	0.1075 (19)	0.614 (7)
F3	0.6535 (12)	0.6952 (7)	0.6034 (6)	0.0961 (19)	0.614 (7)
F4	0.7210 (13)	0.9108 (6)	0.5533 (3)	0.128 (2)	0.614 (7)
F5	0.9725 (6)	0.7933 (8)	0.6592 (6)	0.125 (2)	0.614 (7)
F6	0.800 (3)	0.9748 (11)	0.7254 (10)	0.109 (4)	0.614 (7)
F1'	0.8909 (17)	0.7393 (7)	0.7289 (7)	0.112 (3)	0.386 (7)
F2'	0.5512 (16)	0.7924 (12)	0.6982 (8)	0.140 (4)	0.386 (7)
F3'	0.705 (2)	0.7182 (14)	0.5738 (8)	0.101 (3)	0.386 (7)
F4'	0.5990 (17)	0.9410 (8)	0.5792 (9)	0.127 (4)	0.386 (7)
F5'	0.9347 (16)	0.8884 (11)	0.6106 (7)	0.120 (3)	0.386 (7)
F6'	0.830 (4)	0.9820 (15)	0.7120 (14)	0.084 (4)	0.386 (7)

### 4-[(E)-2-(4-Methoxyphenyl)ethenyl]-1-methylpyridin-1-ium hexafluoro-λ6-phosphane (I) Atomic displacement parameters (Å^2^)

**Table:**

	*U*^11^	*U*^22^	*U*^33^	*U*^12^	*U*^13^	*U*^23^
C1	0.0625 (16)	0.083 (3)	0.0841 (18)	−0.0033 (15)	−0.0058 (14)	0.0087 (17)
C2	0.0537 (15)	0.0581 (17)	0.0568 (14)	−0.0038 (12)	0.0116 (11)	0.0032 (12)
C3	0.0628 (16)	0.0509 (17)	0.0749 (16)	−0.0001 (12)	0.0080 (13)	−0.0089 (13)
C4	0.0593 (15)	0.0527 (16)	0.0669 (14)	0.0059 (12)	0.0070 (12)	−0.0023 (13)
C5	0.0503 (12)	0.0519 (17)	0.0473 (10)	−0.0017 (10)	0.0130 (9)	0.0013 (10)
C6	0.0586 (15)	0.0464 (14)	0.0637 (14)	−0.0030 (12)	0.0145 (12)	−0.0061 (12)
C7	0.0516 (14)	0.0518 (18)	0.0697 (15)	0.0023 (10)	0.0114 (11)	0.0056 (12)
C8	0.0580 (12)	0.0529 (15)	0.0515 (11)	0.0006 (13)	0.0147 (10)	0.0023 (13)
C9	0.0562 (14)	0.0503 (15)	0.0651 (13)	−0.0013 (11)	0.0116 (11)	0.0012 (12)
C10	0.0523 (13)	0.0486 (19)	0.0512 (11)	−0.0008 (10)	0.0157 (9)	0.0026 (10)
C11	0.0652 (17)	0.0521 (16)	0.0623 (14)	0.0028 (13)	0.0113 (12)	−0.0033 (13)
C12	0.0594 (16)	0.0594 (19)	0.0648 (15)	0.0099 (13)	0.0123 (13)	0.0080 (13)
C13	0.0645 (16)	0.0513 (17)	0.0620 (14)	−0.0023 (13)	0.0129 (13)	−0.0010 (13)
C14	0.0569 (16)	0.0503 (17)	0.0700 (16)	0.0082 (12)	0.0144 (13)	0.0036 (13)
C15	0.0552 (15)	0.097 (2)	0.0656 (15)	−0.0093 (15)	0.0013 (12)	0.0037 (16)
N1	0.0497 (11)	0.0629 (19)	0.0507 (10)	−0.0013 (9)	0.0112 (8)	0.0030 (9)
O1	0.0622 (11)	0.0680 (14)	0.0816 (12)	−0.0055 (10)	−0.0061 (9)	−0.0044 (11)
P1	0.0518 (3)	0.0467 (3)	0.0587 (3)	0.0013 (3)	0.0066 (2)	−0.0026 (3)
F1	0.172 (5)	0.089 (3)	0.079 (2)	0.006 (3)	0.017 (3)	0.028 (2)
F2	0.0611 (19)	0.140 (4)	0.113 (3)	0.024 (2)	−0.0018 (19)	−0.038 (3)
F3	0.095 (4)	0.068 (3)	0.116 (4)	−0.008 (2)	−0.001 (3)	−0.025 (3)
F4	0.213 (6)	0.109 (4)	0.073 (2)	0.015 (4)	0.057 (3)	0.015 (2)
F5	0.063 (2)	0.139 (5)	0.173 (5)	0.018 (2)	0.019 (3)	−0.057 (4)
F6	0.111 (5)	0.091 (6)	0.121 (6)	0.004 (4)	0.018 (4)	−0.057 (5)
F1'	0.136 (6)	0.074 (4)	0.099 (5)	0.026 (4)	−0.037 (4)	0.005 (4)
F2'	0.115 (6)	0.149 (7)	0.182 (7)	−0.035 (5)	0.092 (5)	−0.004 (6)
F3'	0.116 (7)	0.093 (6)	0.092 (5)	−0.020 (4)	0.018 (4)	−0.039 (4)
F4'	0.127 (6)	0.080 (4)	0.136 (7)	0.007 (4)	−0.064 (5)	0.017 (4)
F5'	0.115 (5)	0.120 (6)	0.146 (6)	−0.027 (4)	0.079 (5)	−0.019 (5)
F6'	0.102 (7)	0.056 (5)	0.091 (5)	−0.021 (5)	0.010 (5)	−0.009 (4)

### 4-[(E)-2-(4-Methoxyphenyl)ethenyl]-1-methylpyridin-1-ium hexafluoro-λ6-phosphane (I) Geometric parameters (Å, º)

**Table:**

C1—O1	1.418 (4)	C11—H11	0.9300
C1—H1A	0.9600	C12—N1	1.341 (4)
C1—H1B	0.9600	C12—H12	0.9300
C1—H1C	0.9600	C13—N1	1.338 (3)
C2—O1	1.357 (3)	C13—C14	1.358 (4)
C2—C3	1.370 (4)	C13—H13	0.9300
C2—C7	1.382 (4)	C14—H14	0.9300
C3—C4	1.376 (4)	C15—N1	1.470 (3)
C3—H3	0.9300	C15—H15A	0.9600
C4—C5	1.395 (4)	C15—H15B	0.9600
C4—H4	0.9300	C15—H15C	0.9600
C5—C6	1.385 (4)	P1—F2'	1.515 (6)
C5—C8	1.452 (3)	P1—F4	1.537 (4)
C6—C7	1.391 (4)	P1—F5	1.543 (4)
C6—H6	0.9300	P1—F1'	1.545 (6)
C7—H7	0.9300	P1—F4'	1.566 (6)
C8—C9	1.312 (4)	P1—F2	1.566 (4)
C8—H8	0.9300	P1—F1	1.570 (4)
C9—C10	1.458 (4)	P1—F6	1.570 (8)
C9—H9	0.9300	P1—F5'	1.571 (6)
C10—C11	1.390 (4)	P1—F6'	1.575 (12)
C10—C14	1.396 (4)	P1—F3	1.586 (6)
C11—C12	1.352 (4)	P1—F3'	1.588 (10)
			
O1—C1—H1A	109.5	F2'—P1—F1'	91.7 (6)
O1—C1—H1B	109.5	F4—P1—F1'	140.3 (5)
H1A—C1—H1B	109.5	F5—P1—F1'	48.9 (4)
O1—C1—H1C	109.5	F2'—P1—F4'	91.4 (6)
H1A—C1—H1C	109.5	F5—P1—F4'	127.8 (6)
H1B—C1—H1C	109.5	F1'—P1—F4'	176.6 (7)
O1—C2—C3	115.2 (3)	F4—P1—F2	88.8 (3)
O1—C2—C7	125.2 (3)	F5—P1—F2	178.8 (4)
C3—C2—C7	119.7 (2)	F1'—P1—F2	129.9 (5)
C2—C3—C4	121.0 (3)	F4'—P1—F2	53.4 (5)
C2—C3—H3	119.5	F2'—P1—F1	55.5 (5)
C4—C3—H3	119.5	F4—P1—F1	178.2 (4)
C3—C4—C5	121.0 (3)	F5—P1—F1	89.4 (4)
C3—C4—H4	119.5	F4'—P1—F1	142.0 (6)
C5—C4—H4	119.5	F2—P1—F1	89.4 (3)
C6—C5—C4	117.0 (2)	F2'—P1—F6	97.4 (7)
C6—C5—C8	119.9 (2)	F4—P1—F6	99.3 (6)
C4—C5—C8	123.1 (2)	F5—P1—F6	95.9 (7)
C5—C6—C7	122.3 (3)	F1'—P1—F6	94.1 (6)
C5—C6—H6	118.8	F4'—P1—F6	87.2 (7)
C7—C6—H6	118.8	F2—P1—F6	83.7 (6)
C2—C7—C6	118.9 (2)	F1—P1—F6	80.3 (6)
C2—C7—H7	120.5	F2'—P1—F5'	178.3 (5)
C6—C7—H7	120.5	F4—P1—F5'	55.6 (4)
C9—C8—C5	126.3 (3)	F1'—P1—F5'	89.4 (5)
C9—C8—H8	116.8	F4'—P1—F5'	87.5 (6)
C5—C8—H8	116.8	F2—P1—F5'	139.4 (5)
C8—C9—C10	126.5 (3)	F1—P1—F5'	126.0 (5)
C8—C9—H9	116.7	F6—P1—F5'	83.8 (6)
C10—C9—H9	116.7	F2'—P1—F6'	108.1 (9)
C11—C10—C14	116.5 (2)	F4—P1—F6'	91.5 (7)
C11—C10—C9	119.2 (2)	F5—P1—F6'	88.7 (10)
C14—C10—C9	124.2 (2)	F1'—P1—F6'	95.5 (8)
C12—C11—C10	120.5 (3)	F4'—P1—F6'	85.1 (8)
C12—C11—H11	119.7	F2—P1—F6'	91.0 (9)
C10—C11—H11	119.7	F1—P1—F6'	88.3 (7)
N1—C12—C11	121.7 (3)	F5'—P1—F6'	73.1 (9)
N1—C12—H12	119.1	F2'—P1—F3	71.4 (4)
C11—C12—H12	119.1	F4—P1—F3	89.7 (3)
N1—C13—C14	121.5 (3)	F5—P1—F3	91.1 (3)
N1—C13—H13	119.3	F1'—P1—F3	83.5 (4)
C14—C13—H13	119.3	F4'—P1—F3	96.0 (4)
C13—C14—C10	120.4 (3)	F2—P1—F3	89.1 (3)
C13—C14—H14	119.8	F1—P1—F3	90.5 (3)
C10—C14—H14	119.8	F6—P1—F3	168.4 (6)
N1—C15—H15A	109.5	F5'—P1—F3	107.5 (4)
N1—C15—H15B	109.5	F6'—P1—F3	178.8 (8)
H15A—C15—H15B	109.5	F2'—P1—F3'	92.4 (5)
N1—C15—H15C	109.5	F4—P1—F3'	71.7 (5)
H15A—C15—H15C	109.5	F5—P1—F3'	79.2 (5)
H15B—C15—H15C	109.5	F1'—P1—F3'	89.7 (6)
C13—N1—C12	119.3 (2)	F4'—P1—F3'	88.5 (7)
C13—N1—C15	121.0 (3)	F2—P1—F3'	101.4 (5)
C12—N1—C15	119.8 (2)	F1—P1—F3'	108.9 (5)
C2—O1—C1	118.6 (3)	F6—P1—F3'	169.4 (7)
F2'—P1—F4	123.0 (5)	F5'—P1—F3'	86.3 (5)
F2'—P1—F5	139.2 (6)	F6'—P1—F3'	158.7 (9)
F4—P1—F5	92.4 (4)		
			
O1—C2—C3—C4	−179.0 (2)	C8—C9—C10—C14	2.9 (4)
C7—C2—C3—C4	1.8 (4)	C14—C10—C11—C12	0.4 (4)
C2—C3—C4—C5	0.0 (4)	C9—C10—C11—C12	−178.7 (2)
C3—C4—C5—C6	−1.6 (4)	C10—C11—C12—N1	−0.1 (4)
C3—C4—C5—C8	176.3 (2)	N1—C13—C14—C10	−0.6 (4)
C4—C5—C6—C7	1.5 (4)	C11—C10—C14—C13	0.0 (4)
C8—C5—C6—C7	−176.5 (2)	C9—C10—C14—C13	179.0 (2)
O1—C2—C7—C6	179.0 (2)	C14—C13—N1—C12	0.9 (4)
C3—C2—C7—C6	−1.9 (4)	C14—C13—N1—C15	179.1 (3)
C5—C6—C7—C2	0.2 (4)	C11—C12—N1—C13	−0.5 (4)
C6—C5—C8—C9	−179.4 (2)	C11—C12—N1—C15	−178.7 (2)
C4—C5—C8—C9	2.8 (4)	C3—C2—O1—C1	178.2 (3)
C5—C8—C9—C10	−175.8 (2)	C7—C2—O1—C1	−2.7 (4)
C8—C9—C10—C11	−178.1 (2)		

### 4-[(E)-2-(4-Methoxyphenyl)ethenyl]-1-methylpyridin-1-ium hexafluoro-λ6-phosphane (I) Hydrogen-bond geometry (Å, º)

**Table:**

*D*—H···*A*	*D*—H	H···*A*	*D*···*A*	*D*—H···*A*
C1—H1*C*···F2′^i^	0.96	2.51	3.220 (8)	131
C12—H12···F3^ii^	0.93	2.60	3.509 (8)	165
C12—H12···F3′^ii^	0.93	2.53	3.454 (16)	176
C13—H13···F5′^iii^	0.93	2.40	3.270 (9)	156
C15—H15*A*···F4^iii^	0.96	2.53	3.443 (7)	160
C15—H15*B*···F2^iv^	0.96	2.46	3.235 (5)	138
C15—H15*B*···F5′^v^	0.96	2.49	3.162 (7)	127

Symmetry codes: (i) −*x*+1, *y*−1/2, −*z*+1; (ii) *x*−2, *y*, *z*−1; (iii) *x*−2, *y*−1, *z*−1; (iv) −*x*−1, *y*−1/2, −*z*; (v) −*x*, *y*−1/2, −*z*.

### 4-{(E)-2-[4-(Dimethylamino)phenyl]ethenyl}-1-phenyl-1λ5-pyridin-\ 1-ylium hexafluoro-λ6-phosphane (II) Crystal data

**Table:**

C_21_H_21_N_2_^+^·PF_6_^−^	*F*(000) = 920
*M**_r_* = 446.37	*D*_x_ = 1.463 Mg m^−^^3^
Monoclinic, *C**c*	Mo *K*α radiation, λ = 0.71073 Å
Hall symbol: C -2yc	Cell parameters from 3926 reflections
*a* = 19.4596 (14) Å	θ = 2.3–26.0°
*b* = 10.7416 (8) Å	µ = 0.20 mm^−^^1^
*c* = 11.9654 (9) Å	*T* = 296 K
β = 125.864 (2)°	Block, yellow
*V* = 2026.9 (3) Å^3^	0.35 × 0.30 × 0.30 mm
*Z* = 4	

### 4-{(E)-2-[4-(Dimethylamino)phenyl]ethenyl}-1-phenyl-1λ5-pyridin-\ 1-ylium hexafluoro-λ6-phosphane (II) Data collection

**Table:**

Bruker Kappa APEXII CCD diffractometer	3926 independent reflections
Radiation source: fine-focus sealed tube	2895 reflections with *I* > 2σ(*I*)
Graphite monochromator	*R*_int_ = 0.024
φ & ω scans	θ_max_ = 26.0°, θ_min_ = 2.3°
Absorption correction: multi-scan (SADABS; Bruker, 2008)	*h* = −23→23
*T*_min_ = 0.933, *T*_max_ = 0.943	*k* = −13→13
13796 measured reflections	*l* = −14→14

### 4-{(E)-2-[4-(Dimethylamino)phenyl]ethenyl}-1-phenyl-1λ5-pyridin-\ 1-ylium hexafluoro-λ6-phosphane (II) Refinement

**Table:**

Refinement on *F*^2^	Secondary atom site location: difference Fourier map
Least-squares matrix: full	Hydrogen site location: inferred from neighbouring sites
*R*[*F*^2^ > 2σ(*F*^2^)] = 0.064	H atoms treated by a mixture of independent and constrained refinement
*wR*(*F*^2^) = 0.203	*w* = 1/[σ^2^(*F*_o_^2^) + (0.1113*P*)^2^ + 2.2166*P*] where *P* = (*F*_o_^2^ + 2*F*_c_^2^)/3
*S* = 1.07	(Δ/σ)_max_ < 0.001
3926 reflections	Δρ_max_ = 0.49 e Å^−^^3^
255 parameters	Δρ_min_ = −0.41 e Å^−^^3^
65 restraints	Absolute structure: Flack (1983), 1927 Fridel pairs
Primary atom site location: structure-invariant direct methods	Absolute structure parameter: 0.5 (2)

### 4-{(E)-2-[4-(Dimethylamino)phenyl]ethenyl}-1-phenyl-1λ5-pyridin-\ 1-ylium hexafluoro-λ6-phosphane (II) Special details

**Table:**

Geometry. All esds (except the esd in the dihedral angle between two l.s. planes) are estimated using the full covariance matrix. The cell esds are taken into account individually in the estimation of esds in distances, angles and torsion angles; correlations between esds in cell parameters are only used when they are defined by crystal symmetry. An approximate (isotropic) treatment of cell esds is used for estimating esds involving l.s. planes.
Refinement. Refinement of F^2^ against ALL reflections. The weighted R-factor wR and goodness of fit S are based on F^2^, conventional R-factors R are based on F, with F set to zero for negative F^2^. The threshold expression of F^2^ > 2sigma(F^2^) is used only for calculating R-factors(gt) etc. and is not relevant to the choice of reflections for refinement. R-factors based on F^2^ are statistically about twice as large as those based on F, and R- factors based on ALL data will be even larger.

### 4-{(E)-2-[4-(Dimethylamino)phenyl]ethenyl}-1-phenyl-1λ5-pyridin-\ 1-ylium hexafluoro-λ6-phosphane (II) Fractional atomic coordinates and isotropic or equivalent isotropic displacement parameters (Å^2^)

**Table:**

	*x*	*y*	*z*	*U*_iso_*/*U*_eq_	
C1	0.0124 (4)	0.0746 (5)	0.2218 (7)	0.0672 (15)	
H1	−0.0060	0.0194	0.1495	0.081*	
C2	−0.0338 (4)	0.0925 (6)	0.2738 (7)	0.0755 (18)	
H2	−0.0856	0.0520	0.2332	0.091*	
C3	−0.0043 (4)	0.1701 (6)	0.3865 (8)	0.0776 (17)	
H3	−0.0345	0.1785	0.4242	0.093*	
C4	0.0694 (4)	0.2339 (5)	0.4407 (7)	0.0746 (16)	
H4	0.0884	0.2868	0.5149	0.090*	
C5	0.1166 (3)	0.2228 (5)	0.3898 (6)	0.0621 (13)	
H5	0.1663	0.2681	0.4274	0.075*	
C6	0.0876 (3)	0.1414 (5)	0.2800 (5)	0.0537 (12)	
C7	0.1625 (3)	0.0050 (5)	0.2239 (5)	0.0556 (12)	
H7	0.1460	−0.0616	0.2530	0.067*	
C8	0.2108 (3)	−0.0160 (5)	0.1788 (5)	0.0548 (12)	
H8	0.2272	−0.0971	0.1778	0.066*	
C9	0.2369 (3)	0.0807 (5)	0.1335 (5)	0.0535 (12)	
C10	0.2116 (3)	0.2004 (5)	0.1416 (5)	0.0600 (13)	
H10	0.2280	0.2682	0.1140	0.072*	
C11	0.1632 (3)	0.2204 (5)	0.1894 (5)	0.0586 (13)	
H11	0.1482	0.3010	0.1957	0.070*	
C12	0.2873 (3)	0.0638 (5)	0.0829 (5)	0.0569 (12)	
H12	0.3022	0.1343	0.0566	0.068*	
C13	0.3147 (3)	−0.0474 (5)	0.0709 (5)	0.0560 (12)	
H13	0.2995	−0.1165	0.0990	0.067*	
C14	0.3642 (3)	−0.0719 (5)	0.0201 (5)	0.0541 (12)	
C15	0.3748 (4)	−0.1927 (5)	−0.0088 (6)	0.0614 (13)	
H15	0.3505	−0.2577	0.0080	0.074*	
C16	0.4201 (4)	−0.2204 (5)	−0.0616 (6)	0.0656 (14)	
H16	0.4237	−0.3024	−0.0826	0.079*	
C17	0.4603 (3)	−0.1265 (5)	−0.0834 (6)	0.0600 (13)	
C18	0.4494 (3)	−0.0039 (5)	−0.0559 (6)	0.0626 (13)	
H18	0.4744	0.0610	−0.0715	0.075*	
C19	0.4029 (3)	0.0225 (5)	−0.0064 (5)	0.0591 (13)	
H19	0.3967	0.1050	0.0100	0.071*	
C20	0.5037 (5)	−0.2756 (7)	−0.1855 (8)	0.091 (2)	
H20A	0.5358	−0.3311	−0.1083	0.137*	
H20B	0.5284	−0.2734	−0.2353	0.137*	
H20C	0.4462	−0.3045	−0.2453	0.137*	
C21	0.5524 (4)	−0.0558 (7)	−0.1456 (7)	0.0751 (16)	
H21A	0.5151	0.0107	−0.2024	0.113*	
H21B	0.5792	−0.0895	−0.1855	0.113*	
H21C	0.5950	−0.0244	−0.0550	0.113*	
N1	0.5049 (3)	−0.1511 (5)	−0.1362 (5)	0.0724 (13)	
N2	0.1375 (2)	0.1224 (4)	0.2273 (4)	0.0515 (10)	
P1	0.26522 (10)	−0.40739 (11)	0.33958 (15)	0.0597 (4)	
F5	0.1834 (3)	−0.4680 (5)	0.2131 (5)	0.1541 (16)	
F4	0.3249 (3)	−0.5071 (4)	0.3457 (7)	0.1356 (14)	
F3	0.3451 (3)	−0.3462 (6)	0.4685 (5)	0.1541 (16)	
F2	0.2065 (3)	−0.3017 (4)	0.3315 (7)	0.1356 (14)	
F6	0.2762 (3)	−0.3260 (6)	0.2444 (6)	0.1486 (17)	
F1	0.2515 (3)	−0.4916 (5)	0.4289 (6)	0.1486 (17)	

### 4-{(E)-2-[4-(Dimethylamino)phenyl]ethenyl}-1-phenyl-1λ5-pyridin-\ 1-ylium hexafluoro-λ6-phosphane (II) Atomic displacement parameters (Å^2^)

**Table:**

	*U*^11^	*U*^22^	*U*^33^	*U*^12^	*U*^13^	*U*^23^
C1	0.054 (3)	0.051 (3)	0.085 (4)	0.003 (2)	0.034 (3)	0.011 (3)
C2	0.063 (4)	0.061 (4)	0.097 (5)	0.006 (3)	0.044 (4)	0.024 (3)
C3	0.076 (4)	0.067 (4)	0.104 (5)	0.022 (3)	0.060 (4)	0.023 (4)
C4	0.069 (4)	0.058 (3)	0.090 (4)	0.019 (3)	0.043 (3)	0.002 (3)
C5	0.054 (3)	0.049 (3)	0.068 (3)	0.010 (2)	0.028 (3)	0.003 (2)
C6	0.050 (3)	0.042 (2)	0.060 (3)	0.004 (2)	0.027 (2)	0.009 (2)
C7	0.065 (3)	0.043 (3)	0.051 (3)	−0.007 (2)	0.029 (2)	0.004 (2)
C8	0.063 (3)	0.043 (3)	0.058 (3)	−0.007 (2)	0.035 (2)	0.002 (2)
C9	0.052 (3)	0.051 (3)	0.042 (2)	−0.002 (2)	0.019 (2)	0.0032 (19)
C10	0.059 (3)	0.049 (3)	0.061 (3)	−0.008 (2)	0.029 (3)	0.012 (2)
C11	0.058 (3)	0.043 (3)	0.061 (3)	0.002 (2)	0.027 (3)	0.007 (2)
C12	0.057 (3)	0.059 (3)	0.048 (2)	−0.011 (2)	0.027 (2)	0.006 (2)
C13	0.053 (3)	0.053 (3)	0.050 (3)	−0.006 (2)	0.024 (2)	0.005 (2)
C14	0.046 (3)	0.050 (3)	0.049 (2)	−0.006 (2)	0.018 (2)	0.003 (2)
C15	0.058 (3)	0.044 (3)	0.065 (3)	−0.006 (2)	0.027 (3)	0.001 (2)
C16	0.063 (3)	0.039 (3)	0.074 (3)	0.003 (2)	0.029 (3)	0.001 (2)
C17	0.054 (3)	0.048 (3)	0.056 (3)	0.002 (2)	0.020 (2)	−0.005 (2)
C18	0.062 (3)	0.047 (3)	0.076 (3)	−0.014 (2)	0.039 (3)	−0.009 (2)
C19	0.069 (3)	0.042 (3)	0.070 (3)	−0.008 (2)	0.042 (3)	−0.009 (2)
C20	0.085 (4)	0.081 (4)	0.096 (5)	0.009 (4)	0.046 (4)	−0.030 (4)
C21	0.063 (3)	0.086 (4)	0.082 (4)	0.003 (3)	0.045 (3)	−0.007 (3)
N1	0.067 (3)	0.058 (3)	0.091 (3)	−0.003 (2)	0.046 (3)	−0.019 (2)
N2	0.050 (2)	0.040 (2)	0.052 (2)	−0.0041 (16)	0.0224 (19)	0.0060 (16)
P1	0.0620 (7)	0.0432 (6)	0.0712 (8)	0.0011 (6)	0.0376 (6)	0.0017 (6)
F5	0.116 (3)	0.133 (3)	0.129 (3)	−0.033 (2)	0.025 (2)	−0.040 (2)
F4	0.150 (3)	0.080 (2)	0.227 (4)	0.0386 (19)	0.139 (3)	0.023 (2)
F3	0.116 (3)	0.133 (3)	0.129 (3)	−0.033 (2)	0.025 (2)	−0.040 (2)
F2	0.150 (3)	0.080 (2)	0.227 (4)	0.0386 (19)	0.139 (3)	0.023 (2)
F6	0.159 (3)	0.155 (3)	0.194 (4)	0.054 (3)	0.138 (3)	0.096 (3)
F1	0.159 (3)	0.155 (3)	0.194 (4)	0.054 (3)	0.138 (3)	0.096 (3)

### 4-{(E)-2-[4-(Dimethylamino)phenyl]ethenyl}-1-phenyl-1λ5-pyridin-\ 1-ylium hexafluoro-λ6-phosphane (II) Geometric parameters (Å, º)

**Table:**

C1—C2	1.375 (9)	C13—H13	0.9300
C1—C6	1.395 (8)	C14—C15	1.389 (7)
C1—H1	0.9300	C14—C19	1.406 (7)
C2—C3	1.391 (10)	C15—C16	1.384 (8)
C2—H2	0.9300	C15—H15	0.9300
C3—C4	1.363 (9)	C16—C17	1.392 (8)
C3—H3	0.9300	C16—H16	0.9300
C4—C5	1.372 (9)	C17—N1	1.366 (7)
C4—H4	0.9300	C17—C18	1.403 (7)
C5—C6	1.393 (7)	C18—C19	1.371 (7)
C5—H5	0.9300	C18—H18	0.9300
C6—N2	1.449 (7)	C19—H19	0.9300
C7—C8	1.350 (7)	C20—N1	1.456 (8)
C7—N2	1.360 (6)	C20—H20A	0.9600
C7—H7	0.9300	C20—H20B	0.9600
C8—C9	1.397 (7)	C20—H20C	0.9600
C8—H8	0.9300	C21—N1	1.428 (8)
C9—C10	1.401 (7)	C21—H21A	0.9600
C9—C12	1.435 (8)	C21—H21B	0.9600
C10—C11	1.375 (8)	C21—H21C	0.9600
C10—H10	0.9300	P1—F1	1.539 (4)
C11—N2	1.353 (6)	P1—F6	1.547 (4)
C11—H11	0.9300	P1—F4	1.550 (4)
C12—C13	1.348 (8)	P1—F3	1.555 (4)
C12—H12	0.9300	P1—F5	1.557 (4)
C13—C14	1.433 (7)	P1—F2	1.574 (4)
			
C2—C1—C6	118.4 (6)	C15—C16—C17	120.5 (5)
C2—C1—H1	120.8	C15—C16—H16	119.8
C6—C1—H1	120.8	C17—C16—H16	119.8
C1—C2—C3	121.0 (6)	N1—C17—C16	121.7 (5)
C1—C2—H2	119.5	N1—C17—C18	121.0 (5)
C3—C2—H2	119.5	C16—C17—C18	117.2 (5)
C4—C3—C2	118.9 (6)	C19—C18—C17	121.6 (5)
C4—C3—H3	120.5	C19—C18—H18	119.2
C2—C3—H3	120.5	C17—C18—H18	119.2
C3—C4—C5	122.5 (6)	C18—C19—C14	121.6 (5)
C3—C4—H4	118.8	C18—C19—H19	119.2
C5—C4—H4	118.8	C14—C19—H19	119.2
C4—C5—C6	117.8 (6)	N1—C20—H20A	109.5
C4—C5—H5	121.1	N1—C20—H20B	109.5
C6—C5—H5	121.1	H20A—C20—H20B	109.5
C5—C6—C1	121.4 (5)	N1—C20—H20C	109.5
C5—C6—N2	119.6 (4)	H20A—C20—H20C	109.5
C1—C6—N2	119.0 (5)	H20B—C20—H20C	109.5
C8—C7—N2	120.8 (5)	N1—C21—H21A	109.5
C8—C7—H7	119.6	N1—C21—H21B	109.5
N2—C7—H7	119.6	H21A—C21—H21B	109.5
C7—C8—C9	121.9 (5)	N1—C21—H21C	109.5
C7—C8—H8	119.1	H21A—C21—H21C	109.5
C9—C8—H8	119.1	H21B—C21—H21C	109.5
C8—C9—C10	115.6 (5)	C17—N1—C21	120.9 (5)
C8—C9—C12	124.3 (5)	C17—N1—C20	120.1 (6)
C10—C9—C12	120.1 (5)	C21—N1—C20	119.0 (5)
C11—C10—C9	121.8 (5)	C11—N2—C7	120.2 (5)
C11—C10—H10	119.1	C11—N2—C6	120.6 (4)
C9—C10—H10	119.1	C7—N2—C6	119.1 (4)
N2—C11—C10	119.7 (5)	F1—P1—F6	177.6 (4)
N2—C11—H11	120.2	F1—P1—F4	89.5 (3)
C10—C11—H11	120.2	F6—P1—F4	90.1 (3)
C13—C12—C9	124.5 (5)	F1—P1—F3	92.2 (3)
C13—C12—H12	117.7	F6—P1—F3	90.1 (3)
C9—C12—H12	117.7	F4—P1—F3	87.7 (3)
C12—C13—C14	127.8 (5)	F1—P1—F5	86.3 (3)
C12—C13—H13	116.1	F6—P1—F5	91.4 (3)
C14—C13—H13	116.1	F4—P1—F5	93.9 (3)
C15—C14—C19	116.1 (5)	F3—P1—F5	177.8 (4)
C15—C14—C13	120.8 (5)	F1—P1—F2	92.9 (3)
C19—C14—C13	123.1 (5)	F6—P1—F2	87.5 (3)
C16—C15—C14	122.9 (5)	F4—P1—F2	177.5 (3)
C16—C15—H15	118.6	F3—P1—F2	91.4 (3)
C14—C15—H15	118.6	F5—P1—F2	87.1 (3)
			
C6—C1—C2—C3	−3.3 (8)	C14—C15—C16—C17	2.4 (8)
C1—C2—C3—C4	3.4 (9)	C15—C16—C17—N1	−179.6 (5)
C2—C3—C4—C5	−1.1 (9)	C15—C16—C17—C18	−2.9 (8)
C3—C4—C5—C6	−1.0 (8)	N1—C17—C18—C19	178.2 (5)
C4—C5—C6—C1	1.0 (7)	C16—C17—C18—C19	1.5 (8)
C4—C5—C6—N2	−177.1 (5)	C17—C18—C19—C14	0.5 (8)
C2—C1—C6—C5	1.1 (8)	C15—C14—C19—C18	−1.0 (7)
C2—C1—C6—N2	179.2 (5)	C13—C14—C19—C18	−179.6 (5)
N2—C7—C8—C9	−0.2 (7)	C16—C17—N1—C21	−173.9 (5)
C7—C8—C9—C10	1.7 (7)	C18—C17—N1—C21	9.5 (8)
C7—C8—C9—C12	−179.2 (5)	C16—C17—N1—C20	6.8 (9)
C8—C9—C10—C11	−0.9 (7)	C18—C17—N1—C20	−169.8 (6)
C12—C9—C10—C11	179.9 (5)	C10—C11—N2—C7	2.9 (7)
C9—C10—C11—N2	−1.4 (8)	C10—C11—N2—C6	179.0 (4)
C8—C9—C12—C13	1.0 (8)	C8—C7—N2—C11	−2.1 (7)
C10—C9—C12—C13	−179.9 (5)	C8—C7—N2—C6	−178.3 (4)
C9—C12—C13—C14	179.2 (5)	C5—C6—N2—C11	−53.1 (6)
C12—C13—C14—C15	−168.5 (5)	C1—C6—N2—C11	128.8 (5)
C12—C13—C14—C19	10.0 (8)	C5—C6—N2—C7	123.1 (5)
C19—C14—C15—C16	−0.4 (8)	C1—C6—N2—C7	−55.0 (6)
C13—C14—C15—C16	178.1 (5)		

### 4-{(E)-2-[4-(Dimethylamino)phenyl]ethenyl}-1-phenyl-1λ5-pyridin-\ 1-ylium hexafluoro-λ6-phosphane (II) Hydrogen-bond geometry (Å, º)

**Table:**

*D*—H···*A*	*D*—H	H···*A*	*D*···*A*	*D*—H···*A*
C8—H8···F6	0.93	2.59	3.486 (8)	162
C11—H11···F5^i^	0.93	2.55	3.363 (8)	146
C16—H16···F4^ii^	0.93	2.59	3.289 (7)	132
C21—H21*B*···F2^iii^	0.96	2.64	3.516 (8)	152

Symmetry codes: (i) *x*, *y*+1, *z*; (ii) *x*, −*y*−1, *z*−1/2; (iii) *x*+1/2, −*y*−1/2, *z*−1/2.
